# Genetic Variants Associated with Lipid Profiles in Chinese Patients with Type 2 Diabetes

**DOI:** 10.1371/journal.pone.0135145

**Published:** 2015-08-07

**Authors:** Xiaomu Kong, Qi Zhao, Xiaoyan Xing, Bo Zhang, Xuelian Zhang, Jing Hong, Wenying Yang

**Affiliations:** 1 Department of Endocrinology, China-Japan Friendship Hospital, Beijing, China; 2 Department of Epidemiology, Tulane School of Public Health and Tropical Medicine, New Orleans, Louisiana, United States of America; University of Catanzaro Magna Graecia, ITALY

## Abstract

Dyslipidemia is a strong risk factor for cardiovascular disease among patients with type 2 diabetes (T2D). The aim of this study was to identify lipid-related genetic variants in T2D patients of Han Chinese ancestry. Among 4,908 Chinese T2D patients who were not taking lipid-lowering medications, single nucleotide polymorphisms (SNPs) in seven genes previously found to be associated with lipid traits in genome-wide association studies conducted in populations of European ancestry (*ABCA1*, *GCKR*, *BAZ1B*, *TOMM40*, *DOCK7*, *HNF1A*, and *HNF4A*) were genotyped. After adjusting for multiple covariates, SNPs in *ABCA1*, *GCKR*, *BAZ1B*, *TOMM40*, and *HNF1A* were identified as significantly associated with triglyceride levels in T2D patients (*P* < 0.05). The associations between the SNPs in *ABCA1* (rs3890182), *GCKR* (rs780094), and *BAZ1B* (rs2240466) remained significant even after correction for multiple testing (*P* = 8.85×10^−3^, 7.88×10^−7^, and 2.03×10^−6^, respectively). *BAZ1B* (rs2240466) also was associated with the total cholesterol level (*P* = 4.75×10^−2^). In addition, SNP rs157580 in *TOMM40* was associated with the low-density lipoprotein cholesterol level (*P* = 6.94×10^−3^). Our findings confirm that lipid-related genetic loci are associated with lipid profiles in Chinese patients with type 2 diabetes.

## Introduction

Dyslipidemia is a common comorbidity of type 2 diabetes (T2D) [1,2]. Substantial evidence has demonstrated that abnormal plasma lipids and lipoprotein concentrations, including elevated levels of total cholesterol (TC), triglycerides (TG), and low-density lipoprotein cholesterol (LDL-C), and decreased high-density lipoprotein cholesterol (HDL-C) contribute to the pathogenesis of atherosclerosis and related cardiovascular diseases, which are the leading causes of morbidity and mortality in T2D patients [2,3].

Although strongly influenced by smoking, diet, physical activity, and other life style factors, the lipid profile is a highly heritable trait. The heritability for the each lipid level has been found to range from 0.28 to 0.78 in twin and family studies [4]. Partly due to differences in genetic susceptibility, individuals with comparable exposure to high risk factors (including abnormal glucose levels, alcoholic consumption, and overweight status, etc.) have different lipid levels. Through genome-wide association studies (GWAS), multiple genomic loci have been reported be associated with lipid levels among general populations, mostly in Caucasians, including *ABCA1* [5,6], *GCKR* [5,7,8,9,10,11], *BAZ1B* [7], *TOMM40* [7], *DOCK7* [7], *HNF1A* [12], and *HNF4A* [12,13,14]. However, their associations with lipid traits remained unclear among individuals with T2D. It is believed there should be greater emphasis of genetic studies in T2D patients, because the TG, TC, and LDL-C levels in these patients are much higher than average, and the HDL-C level is lower, which results in far greater than average risk of cardiovascular disease. Moreover, it is also speculated that lipid-related genetic loci may contribute to the pathogenesis of T2D and the maintenance of the glycemic homeostasis. For example, rs780094 in the intron region of *GCKR* was found to be related to both fasting glucose and TG in an opposing manner [9]. Therefore, it is important to examine the associations of established genetic loci with lipid profiles in T2D patients as well as to explore their contributions to glycemic-related traits.

In the present study, we genotyped single nucleotide polymorphisms (SNPs) from established lipid-related genomic loci and examined for their association with fasting TG, TC, LDL-C, and HDL-C levels as well as glycemic traits, in a large sample of T2D patients who were not taking lipid-lowering medications from the Chinese National Diabetes and Metabolic Disorders Study (DMS) conducted in 2007–2008 [15].

## Methods

### Ethics statement

The study protocol was approved by the Ethics Committee of the China-Japan Friendship Hospital in Beijing and was conducted in accordance with the Helsinki Declaration II. Written informed consent was obtained from all participants before data collection.

### Study participants

All of the study participants were included in the DMS [15]. T2D was defined using the 1999 World Health Organization (WHO) criteria, including fasting plasma glucose (FPG) ≥ 7.0 mmol/l, 2-h oral glucose tolerance test (OGTT) plasma glucose ≥ 11.1 mmol/l, or a self-reported history of T2D. A total of 4,908 T2D patients, who were not taking lipid-lowering medicine, including statins, fibrates, nicotinic acids, and traditional Chinese medicines, were included in the current analysis. Of these 4,908 T2D patients, 2,646 were newly diagnosed in the DMS study.

### Clinical measurements and laboratory methods

Body weight, height, waist circumference (WC), and hip circumference (HC) were measured using standard methods, and body mass index (BMI) was calculated as weight/height^2^ (kg/m^2^). Each participant completed a standard 75-g OGTT after overnight fasting. Blood samples were drawn at 0 minute, 30 minutes, and 2 hours after OGTT for measurement of plasma glucose and serum insulin concentrations. Serum insulin was measured by a double-antibody radioimmunoassay. The homeostasis model assessment for β-cell function (HOMA-B) and insulinogenic indices were calculated to estimate β-cell function, and the homeostasis model assessment for insulin resistance (HOMA-IR) and Matsuda index (ISIm) were used to assess insulin resistance. The formulas were as follows:

HOMA−B = fasting serum insulin × 20 / (FPG – 3.5) (with serum insulin in mU/l and plasma glucose in mmol/l)

Insulinogenic index = (serum insulin at 30 min – fasting serum insulin) / (plasma glucose at 30 min – FPG) (with serum insulin in mU/l and plasma glucose in mmol/l)

HOMA−IR = fasting serum insulin × FPG / 22.5 (with serum insulin in mU/l and plasma glucose in mmol/l)

$$ISIm\;=\;10,000\;/\;{\left(FPG\;\times \;fasting\;serum\;insulin\;\times \;mean\;OGTT\;glucose\;\times \;mean\;OGTT\;insulin\right)}^{1/2}{\left(with\;serum\;insulin\;in\;mU/l\;and\;plasma\;glucose\;in\;mg/dl\right)}^{}$$

### Blood lipid measurements and definitions

Fasting plasma levels of TG, TC, HDL-C, and LDL-C were measured using an automatic biochemical analyzer (Olympus, Tokyo, Japan) according to standard methods. Abnormal lipid profiles were defined using the clinical criteria according to the Chinese Guidelines on Prevention and Treatment of Dyslipidemia in Adults (2007) [16]. The cut-off values for abnormal lipid profiles were: TG ≥ 150 mg/dl (1.70 mmol/l), HDL-C < 40 mg/dl (1.04 mmol/l), LDL-C ≥ 130 mg/dl (3.37 mmol/l), and TC ≥ 200mg/dl (5.18 mmol/l).

### SNP selection and genotyping

We initially searched the GWAS-validated lipid-related genomic loci in the general populations of European ancestry published in and before 2010. Among them, we eventually picked nine novel genomic loci. The associations of these loci with lipid levels and glycemic traits in the Chinese population, especially in patients with T2D, were still unclear. These genomic loci either showed associations with T2D or cardiovascular diseases in Caucasians, or had critical biological function in lipid metabolism or pancreatic β-cells. Therefore, we speculated that these genomic loci could have larger effect on lipid level and cardiovascular complications in T2D patients. Index SNP of each lipid-related genomic loci were selected and genotyped, including rs3890182 in *ABCA1*, rs10889353 in *DOCK7*, rs157580 in *TOMM40*, rs780094 in *GCKR*, rs2650000 near *HNF1A*, rs1800961 in *HNF4A*, rs2228671 in *LDLR*, rs2240466 in *BAZ1B*, and rs28927680 in *BUD13* [5,6,7,8,9,10,11,12,13,14,17].

Genomic DNA samples were isolated from the peripheral blood using a DNA extraction kit. Genotyping was conducted using Illumina GoldenGate Indexing assays (Illumina Inc., San Diego, CA, USA) according to the manufacturer’s instructions. SNPs with a minor allele frequency (MAF) < 1% (rs2228671 in *LDLR* and rs28927680 in *BUD13*) were excluded from the analysis. The average call rate for the remaining seven SNPs was 98.19%. The concordance rate based on 229 genotyping duplicates was 100%. Relevant information for the seven SNPs is listed in S1 Table.

### Statistical analyses

The Hardy—Weinberg equilibrium test was performed for each SNP using a χ^2^ test in the study population. Non-Gaussian distributed quantitative traits were natural logarithmically transformed to normal distributions. An additive genetic model was assumed. A linear regression model was used to test the associations between SNPs and quantitative lipid traits (TG, TC, HDL-C, and LDL-C levels). Two multivariable models were tested: in model 1, age and sex were adjusted as co-variables; and in model 2, age, sex, and BMI were adjusted. To eliminate the potential influence of hypoglycemic treatments on lipid levels, we then conducted a sensitivity analysis by only including the newly diagnosed T2D patients from the present population. Further, participants were divided into groups using the cut-off criteria for abnormal lipid levels reported by the Chinese Guidelines on Prevention and Treatment of Dyslipidemia in Adults (2007) [16]. A logistic regression model was used to test the contribution of the risk allele of SNPs to abnormal lipid levels. In addition to the single SNP test, we also examined the joint effects of TG-associated SNPs on the risk of an abnormal TG level (TG **≥** 150 mg/dl). A genetic risk score for TG-associated SNPs was constructed using the sum of the risk alleles for elevated TG in the single marker analyses in each individual without missing data. The risk for an abnormal TG level was compared among quartiles of the genetic risk score. The Bonferroni method was used to correct for multiple testing. Statistical analyses were performed using SAS (version 9.3; SAS Institute, Cary, NC, USA) and PLINK software (v1.05; http://pngu.mgh.harvard.edu/purcell/plink/).

## Results

The clinical characteristics of the T2D patients, including newly diagnosed patients, who were not taking a lipid-lowering medication, are shown in Table 1. All of the seven analyzed SNPs adhered to Hardy—Weinberg equilibrium in the study population (S1 Table). The MAFs of the genotyped SNPs in the present study were close to those reported for a Han Chinese population from Beijing in the HapMap project (S1 Table).

**Table 1:** Clinical characteristics of the study population.

Trait	Type 2 diabetes patients	Newly diagnosed type 2 diabetes patients
**N**	4,908	2,646
**Male, %**	2,144, 43.68%	1,160, 43.84%
**Age, years**	55.00 [47.00, 64.00]	56.00 [47.00, 64.00]
**Weight, kg**	66.00 [59.00, 75.00]	66.50 [59.00, 75.00]
**BMI, kg/m** ^**2**^	25.56 [23.37, 28.08]	25.78 [23.53, 28.31]
**WC, cm**	88.00 [81.00, 95.00]	88.00 [82.00, 95.00]
**Fasting plasma glucose, mmol/l**	7.35 [6.23, 9.00]	7.32 [6.20, 8.95]
**30-min OGTT glucose, mmol/l**	11.96 [9.90, 14.32]	12.01 [9.91, 14.38]
**2-h OGTT glucose, mmol/l**	13.40 [11.24, 17.02]	13.21 [11.25, 16.80]
**Fasting serum insulin, mU/l**	8.64 [6.03, 12.47]	8.66 [6.02, 12.50]
**30-min OGTT insulin, mU/l**	20.07 [11.48, 36.53]	20.81 [11.84, 37.01]
**2-h OGTT insulin, mU/l**	31.56 [18.30, 59.01]	32.54 [18.46, 59.07]
**HOMA-B (%)**	46.41 [27.66, 76.57]	46.66 [28.57, 76.95]
**Insulinogenic index**	2.30 [0.71, 5.50]	2.38 [0.81, 5.62]
**HOMA-IR**	2.97 [1.92, 4.54]	2.96 [1.89, 4.51]
**ISIm**	4.27 [2.87, 6.29]	4.27 [2.85, 6.39]
**Triglycerides, mg/dl**	142.56 [100.94,210.74]	143.44 [100.94,211.62]
**Total cholesterol, mg/dl**	195.65 [169.75,221.95]	196.43 [170.52,222.33]
**HDL-C, mg/dl**	47.56 [40.60, 56.45]	47.95 [40.99, 56.45]
**LDL-C, mg/dl**	117.55 [96.67,139.20]	117.93 [96.67,139.20]

Abbreviations: BMI, body mass index; HDL-C, high-density lipoprotein cholesterol; HOMA-B, the homeostasis model assessment for β-cell function; HOMA-IR, the homeostasis model assessment for insulin resistance; LDL-C, low-density lipoprotein cholesterol; OGTT, oral glucose tolerance test; ISIm, Matsuda index; WC, waist circumference. Data are shown as median (interquartile range) or %.

### Associations of SNPs with lipid profiles in T2D patients of Chinese ancestry

As shown in Table 2, we identified the G allele of rs3890182 in *ABCA1*, the A allele of rs157580 in *TOMM40*, the A allele of rs780094 in *GCKR*, the T allele of rs2650000 near *HNF1A*, and the C allele of rs2240466 in *BAZ1B* as significantly associated with elevated TG levels independent of BMI (*P* = 7.88×10^−7^ to 4.23×10^−2^). The associations of *ABCA1*, *GCKR*, and *BAZ1B* with the TG level were significant even after correction for multiple testing using the Bonferroni method (*P* < 7.14×10^−3^). We also found that the A allele of rs780094 in *GCKR* was associated with an elevated TC level (*P* = 3.50×10^−2^). The C allele of rs2240466 in *BAZ1B* showed nominal association with an elevated TC level after adjustment for BMI (*P* = 4.75×10^−2^). The G allele of rs157580 in *TOMM40* was associated with the LDL-C level (*P* = 7.53×10^−3^), and the association retained significance after adjustment for BMI (*P* = 6.93×10^−3^). No significant effects of these SNPs on the HDL-C level were observed in the current study. When we examined the associations of SNPs and lipid levels in newly diagnosed T2D patients to eliminate the effect of glucose-lowering treatment, the associations of *ABCA1*, *TOMM40*, *GCKR*, and *BAZ1B* with TG levels were confirmed (S2 Table).

**Table 2:** Associations between SNPs and lipid levels among Chinese T2D patients not taking a lipid-lowering medication.

Trait	SNP	Gene	Chr.	Major/minor allele	Model 1		Model 2	
					*β* (SE)	*P*	*β* (SE)	*P*
**TG**	rs3890182	*ABCA1*	9	G/A	-0.029 (0.011)	**5.57×10** ^**−3**^	-0.027 (0.010)	**8.85×10** ^**−3**^
	rs10889353	*DOCK7*	1	A/C	-0.008 (0.007)	2.42×10^−1^	-0.007 (0.007)	3.06×10^−1^
	rs157580	*TOMM40*	19	G/A	0.013 (0.005)	**1.38×10** ^**−2**^	0.013 (0.005)	**1.07×10** ^**−2**^
	rs780094	*GCKR*	2	A/G	-0.025 (0.005)	**1.15×10** ^**−6**^	-0.025 (0.005)	**7.88×10** ^**−7**^
	rs2650000	*HNF1A*	12	G/T	0.010 (0.005)	**4.23×10** ^**−2**^	0.010 (0.005)	**3.85×10** ^**−2**^
	rs1800961	*HNF4A*	20	C/T	0.004 (0.019)	8.32×10^−1^	0.004 (0.018)	8.21×10^−1^
	rs2240466	*BAZ1B*	7	C/T	-0.031 (0.008)	**4.19×10** ^**−5**^	-0.035 (0.007)	**2.03×10** ^**−6**^
**TC**	rs3890182	*ABCA1*	9	G/A	-0.005 (0.004)	2.04×10^−1^	-0.004 (0.004)	2.31×10^−1^
	rs10889353	*DOCK7*	1	A/C	-0.000 (0.003)	9.56×10^−1^	0.000 (0.002)	9.50×10^−1^
	rs157580	*TOMM40*	19	G/A	-0.004 (0.002)	5.48×10^−2^	-0.004 (0.002)	5.80×10^−2^
	rs780094	*GCKR*	2	A/G	-0.004 (0.002)	**3.50×10** ^**−2**^	-0.003 (0.002)	6.15×10^−2^
	rs2650000	*HNF1A*	12	G/T	0.001 (0.002)	5.38×10^−1^	0.001 (0.002)	6.52×10^−1^
	rs1800961	*HNF4A*	20	C/T	-0.009 (0.007)	1.88×10^−1^	-0.009 (0.007)	1.81×10^−1^
	rs2240466	*BAZ1B*	7	C/T	-0.005 (0.003)	9.22×10^−2^	-0.005 (0.003)	**4.75×10** ^**−2**^
**HDL-C**	rs3890182	*ABCA1*	9	G/A	-0.001 (0.005)	8.01×10^−1^	-0.002 (0.005)	7.23×10^−1^
	rs10889353	*DOCK7*	1	A/C	-0.005 (0.003)	1.26×10^−1^	-0.005 (0.003)	1.01×10^−1^
	rs157580	*TOMM40*	19	G/A	0.000 (0.002)	9.25×10^−1^	0.000 (0.002)	9.31×10^−1^
	rs780094	*GCKR*	2	A/G	-0.002 (0.002)	4.62×10^−1^	-0.002 (0.002)	4.64×10^−1^
	rs2650000	*HNF1A*	12	G/T	-0.002 (0.002)	4.63×10^−1^	-0.002 (0.002)	4.68×10^−1^
	rs1800961	*HNF4A*	20	C/T	-0.015 (0.009)	7.39×10^−2^	-0.015 (0.008)	7.13×10^−2^
	rs2240466	*BAZ1B*	7	C/T	-0.000 (0.003)	9.15×10^−1^	0.001 (0.003)	8.14×10^−1^
**LDL-C**	rs3890182	*ABCA1*	9	G/A	-0.008 (0.007)	2.53×10^−1^	-0.007 (0.007)	2.98×10^−1^
	rs10889353	*DOCK7*	1	A/C	0.005 (0.004)	2.78×10^−1^	0.005 (0.004)	2.40×10^−1^
	rs157580	*TOMM40*	19	G/A	-0.009 (0.003)	**7.53×10** ^**−3**^	-0.009 (0.003)	**6.94×10** ^**−3**^
	rs780094	*GCKR*	2	A/G	-0.001 (0.003)	8.15×10^−1^	-0.001 (0.003)	7.97×10^−1^
	rs2650000	*HNF1A*	12	G/T	-0.000 (0.003)	9.97×10^−1^	-0.000 (0.003)	9.98×10^−1^
	rs1800961	*HNF4A*	20	C/T	-0.019 (0.012)	9.62×10^−2^	-0.019 (0.012)	9.44×10^−2^
	rs2240466	*BAZ1B*	7	C/T	0.001 (0.005)	8.54×10^−1^	-0.000 (0.005)	9.66×10^−1^

Abbreviations: BMI, body mass index; Chr, chromosome; HDL-C, high-density lipoprotein cholesterol; LDL-C, low-density lipoprotein cholesterol; SE, standard error; SNP, single nucleotide polymorphism; TC, total cholesterol; TG, triglycerides. All non-Gaussian distributed quantitative traits were natural logarithmically transformed to normalize distributions. *β* value and SE are reported for the minor allele of each SNP using linear regression under an additive assumption using the following models: model 1, adjusted for age and sex; model 2, adjusted for age, sex, and BMI. *P* values <0.05 are shown in bold.

We further defined abnormal lipid levels using the cut-off criteria reported by the Chinese Guidelines on Prevention and Treatment of Dyslipidemia in Adults (2007). As shown in Table 3, SNPs in *ABCA1*, *TOMM40*, *GCKR*, and *BAZ1B* were significantly associated with an abnormal TG level (defined as TG ≥ 150 mg/dl [16]). SNPs in *GCKR* and *BAZ1B* were found to be significantly associated with an abnormal TC level (defined as TC ≥ 200 mg/dl [16]).

**Table 3:** Associations between SNPs and hyperlipidemia among Chinese T2D patients not taking a lipid-lowering medication.

Type of hyperlipidemia	SNP	Gene	Chr.	Major/minor allele	Model 1		Model 2	
					OR (95%CI)	*P*	OR (95%CI)	*P*
**TG ≥ 150 mg/dl**	rs3890182	*ABCA1*	9	G/A	0.80 (0.68, 0.95)	**1.13×10** ^**−2**^	0.81 (0.68, 0.96)	**1.57×10** ^**−2**^
**TG ≥ 150 mg/dl**	rs157580	*TOMM40*	19	G/A	1.12 (1.03, 1.21)	**7.56×10** ^**−3**^	1.13 (1.04, 1.23)	**3.58×10** ^**−3**^
**TG ≥ 150 mg/dl**	rs780094	*GCKR*	2	A/G	0.85 (0.79, 0.93)	**1.28×10** ^**−4**^	0.86 (0.79, 0.93)	**3.04×10** ^**−4**^
**TG ≥ 150 mg/dl**	rs2240466	*BAZ1B*	7	C/T	0.81 (0.72, 0.92)	**6.84×10** ^**−4**^	0.78 (0.69, 0.88)	**7.97×10** ^**−5**^
**TC ≥ 200 mg/dl**	rs780094	*GCKR*	2	A/G	0.92 (0.85, 1.00)	**4.76×10** ^**−2**^	0.93 (0.85, 1.00)	6.38×10^−2^
**TC ≥ 200 mg/dl**	rs2240466	*BAZ1B*	7	C/T	0.85 (0.75, 0.96)	**7.00×10** ^**−3**^	0.84 (0.74, 0.94)	**3.85×10** ^**−3**^

Abbreviations: BMI, body mass index; Chr, chromosome; CI, confidence interval; OR, odds ratio; SNP, single nucleotide polymorphism; TC, total cholesterol; TG, triglycerides. ORs and 95% CIs were determined for the minor allele of each SNP using logistic regression under an additive assumption using the following models: model 1, adjusted for age and sex; model 2, adjusted for age, sex, and BMI. Associations with *P* values <0.05 are shown in the table and denoted in bold.

### Joint analysis of TG-associated SNPs on TG levels in T2D patients of Chinese ancestry

Joint effect analysis showed that the genetic risk scores for TG-associated SNPs were significantly associated with the TG level, as well as the risk of an abnormal TG level (TG **≥** 150 mg/dl; Table 4). Individuals with more risk alleles had an elevated TG level (Q1~Q4: 129.27 (92.09, 186.83) mg/dl, 142.56 (99.17, 204.54) mg/dl, 149.64 (107.14, 221.36) mg/dl, and 147.87 (103.60, 223.13) mg/dl). Compared to the lowest quartile of the genetic risk score, the ORs (95% CIs) for greater risk of an abnormal TG were 1.27 (1.08, 1.49), 1.49 (1.27, 1.75), and 1.45 (1.22, 1.73) for the other three quartiles (*P* for trend = 5.17×10^−8^). The association remained significant after additional adjustment for BMI (*P* for trend = 3.48×10^−8^).

**Table 4:** Joint effects of TG-related risk alleles on TG level and the risk for a high TG level (≥ 150 mg/dl) in T2D patients.

Quartile	TG, mg/dl	TG ≥ 150 mg/dl	
		OR (95% CI)^c^	OR (95% CI)^d^
**Q1**	129.27 (92.09, 186.83)	1	1
**Q2**	142.56 (99.17, 204.54)	1.27 (1.08,1.49)	1.30 (1.10,1.54)
		*P* = **4.34×10** ^**−3**^	*P* = **1.89×10** ^**−3**^
**Q3**	149.64 (107.14, 221.36)	1.49 (1.27,1.75)	1.51 (1.28,1.78)
		*P* = **1.36×10** ^**−6**^	*P* = **1.08×10** ^**−6**^
**Q4**	147.87 (103.60, 223.13)	1.45 (1.22,1.73)	1.49 (1.25,1.77)
		*P* = **2.10×10** ^**−5**^	*P* = **1.03×10** ^**−5**^
	*P* ^a^ = **1.39×10** ^**−12**^	*P*_trend_ = **5.17×10** ^**−8**^	*P*_trend_ = **3.48×10** ^**−8**^
	*P* ^b^ = **1.71×10** ^**−13**^		

Abbreviations: BMI, body mass index; CI, confidence interval; OR, odds ratio; Q, quartile; TG, triglycerides. All non-Gaussian distributed quantitative traits were natural logarithmically transformed to normalize distributions.

^a^, *P* value calculated for genotype risk score for TG level using linear regression adjusted for age and sex.

^b^, *P* value calculated for genotype risk score for TG level using linear regression adjusted for age, sex, and BMI.

^c^, OR and 95% CI are reported for genotype risk score quartiles for a high TG level (≥ 150 mg/dl) using logistic regression adjusted for age and sex.

^d^, OR and 95% CI are reported for genotype risk score quartiles for a high TG level (≥ 150 mg/dl) using logistic regression adjusted for age, sex, and BMI.

*P* values <0.05 are shown in bold.

### Association of SNPs and glycemic-related traits in T2D patients of Chinese ancestry

As shown in Table 5, the T allele of rs2240466 in *BAZ1B* was associated with increased body weight and BMI in T2D patients. The G allele of rs780094 in *GCKR* was associated with increased fasting plasma glucose and HOMA-IR (assessment for insulin resistance). The T allele of rs1800961 in *HNF4A* was associated with increased FPG and 2-h OGTT glucose and decreased 30-min OGTT insulin and HOMA-B (assessment for β-cell function). When tested in newly diagnosed T2D patients, the association of FPG and *GCKR* as well as the association of HOMA-B and *HNF4A* were confirmed (S3 Table).

**Table 5:** Association between SNPs and obesity and glycemic-related traits among Chinese T2D patients not taking a lipid-lowering medication.

Traits	SNP	Gene	Chr.	Minor allele	Model 1		Model 2	
					*β* (SE)	*P*	*β* (SE)	*P*
**Body weight**	rs2240466	*BAZ1B*	7	T	0.005 (0.002)	**2.22×10** ^**−2**^	/	**/**
**BMI**	rs2240466	*BAZ1B*	7	T	0.005 (0.002)	**6.31×10** ^**−3**^	/	**/**
**FPG**	rs780094	*GCKR*	2	G	0.006 (0.003)	**2.89×10** ^**−2**^	0.006 (0.003)	**2.88×10** ^**−2**^
**FPG**	rs1800961	*HNF4A*	20	T	0.026 (0.010)	**8.89×10** ^**−3**^	0.026 (0.010)	**8.86×10** ^**−3**^
**2-h OGTT glucose**	rs1800961	*HNF4A*	20	T	0.027 (0.012)	**2.99×10** ^**−2**^	0.027 (0.012)	**2.97×10** ^**−2**^
**30-min OGTT insulin**	rs1800961	*HNF4A*	20	T	-0.059 (0.030)	5.06×10^−2^	-0.061 (0.029)	**3.90×10** ^**−2**^
**HOMA-B**	rs1800961	*HNF4A*	20	T	-0.070 (0.029)	**1.43×10** ^**−2**^	-0.073 (0.028)	**1.00×10** ^**−2**^
**HOMA-IR**	rs780094	*GCKR*	2	G	0.012 (0.006)	5.73×10^−2^	0.014 (0.006)	**2.93×10** ^**−2**^

Abbreviations: BMI, body mass index; Chr, chromosome; FPG, fasting plasma glucose; HOMA-B, the homeostasis model assessment for β-cell function; HOMA-IR, the homeostasis model assessment for insulin resistance; OGTT, oral glucose tolerance test; SE, standard error; SNP, single nucleotide polymorphism. All non-Gaussian distributed quantitative traits were natural logarithmically transformed to normalize distributions. *β* values were calculated for the minor allele using linear regression under an additive assumption using the following models: model 1, adjusted for age and sex; model 2, adjusted for age, sex, and BMI. Associations with *P* value <0.05 are shown in the table and denoted in bold.

## Discussion

The present study examined the associations of seven SNPs in previously GWAS-identified lipid-related genomic loci in general populations with plasma lipid levels in Chinese T2D patients. Our results showed that five SNPs from *GCKR*, *BAZ1B*, *ABCA1*, *TOMM40*, and *HNF1A* were significantly associated with the fasting TG level individually and jointly. In addition, SNPs in *GCKR* and *BAZ1B* showed nominal associations with the fasting TC level and the SNP in *TOMM40* was associated with the fasting LDL-C level in T2D patients.

Dyslipidemia is a particularly common phenotype in diabetes patients that is characterized by hypertriglyceridemia, reduced HDL-C, and increased LDL-C [1,2]. In addition, epidemic findings have showed that the spectrum of dyslipidemia in diabetes patients can include all of the various types of dyslipidemia identified in general populations. However, compared to the general population, patients with diabetes have significantly elevated TG, TC, and LDL-C levels and a reduced HDL-C level. The greater risk for diabetes complications can be induced by lipid disorders under hyperglycemic condition. Therefore, it is worthwhile to study the mechanism underlying the pathogenesis of dyslipidemia in T2D. Genetic studies have demonstrated that both diabetes and lipid levels are highly heritable phenotypes. In recent decades, a multitude of susceptible genetic loci have been shown to be associated with lipid levels in the general population [5,6,7,8,9,10,11,12,13,14,17]. However, these findings need to be replicated in T2D patients. Moreover, due to ethnic discrepancies and limited sample sizes in previous studies, these findings need to be replicated in larger populations of different ethnicities. The DMS is a large multi-center nation survey of diabetes and metabolic disorders conducted in mainland China, and the T2D patients included in the present study represent well the Chinese Han population [15].

*GCKR*, which encodes the glucokinase regulator, showed the strongest association with the TG level. The glucokinase regulator acts as inhibitor of glucokinase in both liver and pancreatic islet cells [18]. Rs780094 in the intron of *GCKR* has been established to contribute to various metabolic traits by GWAS conducted in Caucasians, including levels of TG [5,7,8,11], LDL-C [10], fasting glucose and insulin [17,19], uric acid [20], etc. Moreover, it was shown to be associated with TG levels in a GWAS conducted in African Americans [8]. Previous studies including one by our group [21] have established its association with FPG levels in Chinese individuals without diabetes. The current study successfully confirmed that the A allele of rs780094 was associated with an elevated fasting TG level and lower FPG level in a Chinese population of diabetes patients, which is consistent with the findings of previous studies [22,23,24,25,26]. Moreover, the A allele showed a marginal association with an elevated TC level and better insulin sensitivity. These results confirm that *GCKR* plays a critical role in both glucose and lipid metabolism.

The second strongest association was found between rs2240466 in *BAZ1B* and the TG level. *BAZ1B*, which is also known as Williams Syndrome Transcription Factor (WSTF), is deleted in Williams—Beuren syndrome [27]. It encodes a member of the bromodomain protein family, which has functions including chromatin assembly, transcription, and repair [27]. Several variants in this gene region have been shown to be related to the TG level, including the C allele of rs2240466, which contributes to an elevated TG level in Caucasians [7]. The current study confirmed the association between the C allele of rs2240466 and an elevated TG level in our Chinese population, and this association was in the same direction as that observed in Caucasians. Moreover, the present study also showed that C allele carriers exhibited a higher TC level and a lower BMI. These findings suggest the involvement of *BAZ1B* in lipid metabolism. However, the biological function of *BAZ1B* in lipid metabolism remains unclear and requires further elucidation.

*ABCA1* is the coding gene for ATP-binding cassette transporter A1, which functions as a cholesterol transporter and has been suggested to mediate the efflux of cholesterol from cells in the presence of HDL and apolipoprotein A-1 [28]. Homozygous mutations in *ABCA1* cause Tangier disease, a rare HDL-deficiency syndrome [29]. Rs3890182, a common variant located in the intron of *ABCA1*, has been shown to be associated with HDL-C levels in Caucasians using a GWAS approach [5,6]. In our study, A allele carriers of rs3890182 had lower TG levels, but no significant association between HDL-C and rs3890182 was identified.

Rs157580 is located in the intron of the *TOMM40-APOE* genetic loci. *TOMM40* encodes the channel-forming subunit of the transclocase of the mitochondrial outer membrane complex, which is essential for protein import into mitochondria [30]. *APOE* encodes apolipoprotein E (apoE), which mediates the clearance of different lipoproteins from the circulation [31]. ApoE deficiency has been reported to cause significant lipid disorders and cardiovascular disease [31]. Previous GWASs have revealed that rs157580 is associated with Alzheimer’s disease [32] as well as HDL-C and LDL-C levels in Caucasians [7]. In the current study, we observed that the A allele of rs157580 was significantly associated with decreased LDL-C levels, which is consistent with the direction of the association observed in Caucasians. We also found that this SNP was significantly associated with TG and TC levels, consistent with the findings of a previous study conducted in a Chinese population [33]. However, we did not observe any association between this SNP and HDL-C in our study population.

Rs2650000 was mapped to the intergenic region of *RPL12P33-HNF1A-AS1* and was found to be related to LDL-C levels in Caucasians [12]. In this genetic locus, *HNF1A* encodes the hepatocyte nuclear factor-1A, which is crucial for pancreatic β cell function [34,35]. Mutations in *HNF1A* were shown to lead to maturity onset diabetes of the young (MODY), which is known as MODY3 [34,35]. In the present study, the A allele of rs2650000 showed a marginal association with elevated TG levels, but no association between this SNP and the LDL-C level was observed. Another study conducted in a Chinese population reported similar results [33]. These findings need to be further confirmed.

In addition, considering that dyslipidemia is a common phenotype in diabetes patients and that the baseline TG level is an independent predictive risk factor for the incidence of T2D in Chinese individuals [1], we speculated that diabetes and dyslipidemia could share common genetic factors. For example, the G allele of rs780094 in *GCKR* was found to be related to an elevated FPG level and a reduced TG level at the genome-wide level, and these results have been well replicated in various populations, including in the control population of the DMS [21]. Therefore, we examined the association of these SNPs and the risk for T2D in the DMS population [36,37], which consisted of 5,338 T2D cases and 4,663 controls, but no significant association was identified (S4 Table). However, we did confirm the association between *GCKR* and the FPG level in T2D patients. We also identified the association between *HNF4A* and the β-cell function index HOMA-B. These associations retained significance in the sensitivity analysis of only the newly diagnosed T2D patients. The *HNF4A* coding product, hepatocyte nuclear factor-4, is a nuclear transcription factor that plays essential roles in the development of the liver, kidney, and intestines [34,35]. It is also expressed in pancreatic β-cells and is crucial for the development and function of β-cells [34,35]. Mutations in *HNF4A* were found to lead to MODY1 [34,35]. Rs1800961 is a missense variation located in the coding region of *HNF4A*, which was previously identified to be related to TC and HDL-C levels [12,13,14]. In addition to the findings described above, *BAZ1B*, *GCKR*, *HNF4A*, and *DOCK7* were associated with some glycemic-related traits including BMI and glucose and insulin levels. The above findings suggest that lipid-related genetic variants can contribute to glycemic regulation.

The present study has several strengths. First, the study population was a genetically homogeneous Chinese Han population with a large sample size. Second, the study examined the association between lipid-related SNPs and lipid profiles in T2D patients who were not taking a lipid-lowering medication. Moreover, a sensitive analysis was conducted in newly diagnosed patients to exclude the effects of glucose-lowering therapy. Therefore, the results could provide good replication in Chinese patients, and good estimates of the effects of genetic loci on lipid levels in T2D patients. Third, we explored whether lipid-related SNPs could contribute to glycemic traits or the risk for T2D. However, our study had some limitations. First, only seven index SNPs in GWAS-validated genomic loci were investigated. Thus, the association between the other genomic loci and lipid levels in this population should be examined in the future study. Second, only one SNP from each genetic locus was analyzed, which might lead to false-negative findings due to the lack of coverage of genomic regions of interest. Third, further studies may be still necessary to replicate our study findings in Chinese T2D patients. In addition, although we identified genetic variants significantly associated with the lipid profile in T2D patients, the mechanisms of these genetic variants in the regulation of lipid levels are not clear. Further studies investigating the functions of these genetic variants significantly associated with lipid profiles are warranted in the future.

In conclusion, the present study investigated the associations between several lipid-related genetic variants and lipid profiles in a Chinese population of T2D patients. Among the tested SNPs, genetic variants in *GCKR*, *BAZ1B*, *ABCA1*, *TOMM40*, and nearby *HNF1A* were associated with the TG level. Genetic variants in *GCKR* and *BAZ1B* showed a nominal association with the TC level, and a genetic variant in *TOMM40* contributed to the LDL-C level. The present study improves our understanding of lipid regulation in T2D in Chinese patients. In addition, it provides evidence that lipid-related genetic loci may affect glycemic metabolism.

## Supporting Information

S1 Table Hardy—Weinberg equilibrium and information for genotyped SNPs. Abbreviations: BMI, body mass index; Chr, chromosome; EU, European; HB, Han Chinese; HDL-C, high-density lipoprotein cholesterol; LDL-C, low-density lipoprotein cholesterol; MAF, minor allele frequency; SNP, single nucleotide polymorphism; TC, total cholesterol; TG, triglycerides. Genotype distributions are shown as the counts of three genotypes (bb, Bb, BB). b, minor allele; B, major allele.
(DOCX)

S2 Table Associations between SNPs and lipid levels among newly diagnosed Chinese T2D patients not taking a lipid-lowering medication. Abbreviations: BMI, body mass index; Chr, chromosome; HDL-C, high-density lipoprotein cholesterol; LDL-C, low-density lipoprotein cholesterol; SE, standard error; SNP, single nucleotide polymorphism; TC, total cholesterol; TG, triglycerides. All non-Gaussian distributed quantitative traits were natural logarithmically transformed to normalize distributions. *β* value and SE were determined for the minor allele of each SNP using linear regression under an additive assumption using the following models: model 1, adjusted for age and sex; model 2, adjusted for age, sex, and BMI. *P* values <0.05 are shown in bold.
(DOCX)

S3 Table Associations between SNPs and glycemic-related traits among newly diagnosed Chinese T2D patients not taking a lipid-lowering medication. Abbreviations: BMI, body mass index; Chr, chromosome; HOMA-B, the homeostasis model assessment for β-cell function; HOMA-IR, the homeostasis model assessment for insulin resistance; OGTT, oral glucose tolerance test; SE, standard error; SNP, single nucleotide polymorphism. All non-Gaussian distributed quantitative traits were natural logarithmically transformed to normalize distributions. *β* values were calculated for the minor allele using linear regression under an additive assumption using the following models: model 1, adjusted for age and sex; model 2, adjusted for age, sex, and BMI. Associations with a *P* value <0.05 are shown in the table and denoted in bold.
(DOCX)

S4 Table Associations between lipid-related SNPs and T2D in DMS patients. Abbreviations: BMI, body mass index; Chr, chromosome; DMS, Chinese National Diabetes and Metabolic Disorders Study; CI, confidence interval; OR, odds ratio; SNP, single nucleotide polymorphism. The DMS population consisted of 5,338 T2D patients and 4,663 controls as previously described [36,37]. ORs and 95% CIs were calculated for the minor allele of each SNP using logistic regression under an additive assumption using the following models: model 1, adjusted for age and sex; model 2, adjusted for age, sex, and BMI.
(DOCX)
