# Supplementary material for: Genetic Variants Associated with Lipid Profiles in Chinese Patients with Type 2 Diabetes
Source: PLoS One. 2015 Aug 7;10(8):e0135145. doi: 10.1371/journal.pone.0135145 (PMC4529182; doi:10.1371/journal.pone.0135145)
Supplement: S1 Table — Abbreviations: BMI, body mass index; Chr, chromosome; EU, European; HB, Han Chinese; HDL-C, high-density lipoprotein cholesterol; LDL-C, low-density lipoprotein cholesterol; MAF, minor allele frequency; SNP, single nucleotide polymorphism; TC, total cholesterol; TG, triglycerides. Genotype distributions are shown as the counts of three genotypes (bb, Bb, BB). b, minor allele; B, major allele. (DOCX) [file pone.0135145.s001.docx]

**S1 Table. Hardy–Weinberg equilibrium and information for genotyped SNPs.**

| **SNP** | **Gene** | **Full gene name** | **Chr.** | **Position (build 38)** | **Localization** | **Major/minor allele** | **MAF** | **Genotype** | ***P*_HWE_** | **Hapmap reported MAF** | | **Related traits reported*** |
| --- | --- | --- | --- | --- | --- | --- | --- | --- | --- | --- | --- | --- |
|  |  |  |  |  |  |  |  | **(bb/Bb/BB)** |  | **EU** | **HB** |  |
| rs3890182 | *ABCA1* | ATP-binding cassette, sub-family A, member 1 | 9 | 104885374 | Intron | G/A | 0.062 | 18/571/4297 | 1.000 | 0.080 | 0.066 | HDL-C |
| rs10889353 | *DOCK7* | Dedicator of cytokinesis 7 | 1 | 62652525 | Intron | A/C | 0.183 | 135/1380/3004 | 0.135 | 0.336 | 0.175 | TG, TC, LDL-C |
| rs157580 | *TOMM40* | Translocase of outer mitochondrial membrane 40 homolog | 19 | 44892009 | Intron | G/A | 0.437 | 912/2407/1526 | 0.502 | 0.628 | 0.449 | HDL-C, LDL-C |
| rs780094 | *GCKR* | Glucokinase regulator | 2 | 27518370 | Intron | A/G | 0.492 | 1167/2445/1250 | 0.688 | 0.606 | 0.434 | TG, LDL-C, FPG |
| rs2650000 | *HNF1A* | Hepatocyte nuclear factor-1 homeobox A | 12 | 120951159 | Intergenic | G/T | 0.480 | 1127/2412/1323 | 0.667 | 0.363 | 0.442 | LDL-C |
| rs1800961 | *HNF4A* | Hepatocyte nuclear factor 4 | 20 | 44413724 | Coding | C/T | 0.018 | 4/172/4721 | 0.082 | 0.018 | 0.023 | TC, HDL-C |
| rs2240466 | *BAZ1B* | Bromodomain adjacent to zinc finger domain, 1B | 7 | 73441939 | Intron | C/T | 0.134 | 78/1143/3643 | 0.293 | 0.121 | 0.142 | TG |

Abbreviations: BMI, body mass index; Chr, chromosome; EU, European; HB, Han Chinese; HDL-C, high-density lipoprotein cholesterol; LDL-C, low-density lipoprotein cholesterol; MAF, minor allele frequency; SNP, single nucleotide polymorphism; TC, total cholesterol; TG, triglycerides.

Genotype distributions are shown as the counts of three genotypes (bb, Bb, BB). b, minor allele; B, major allele.
