# Supplementary material for: Genetic Variants Associated with Lipid Profiles in Chinese Patients with Type 2 Diabetes
Source: PLoS One. 2015 Aug 7;10(8):e0135145. doi: 10.1371/journal.pone.0135145 (PMC4529182; doi:10.1371/journal.pone.0135145)
Supplement: S2 Table — Abbreviations: BMI, body mass index; Chr, chromosome; HDL-C, high-density lipoprotein cholesterol; LDL-C, low-density lipoprotein cholesterol; SE, standard error; SNP, single nucleotide polymorphism; TC, total cholesterol; TG, triglycerides. All non-Gaussian distributed quantitative traits were natural logarithmically transformed to normalize distributions. β value and SE were determined for the minor allele of each SNP using linear regression under an additive assumption using the following models: model 1, adjusted for age and sex; model 2, adjusted for age, sex, and BMI. P values <0.05 are shown in bold. (DOCX) [file pone.0135145.s002.docx]

**S2 Table. Associations between SNPs and lipid levels among newly diagnosed Chinese T2D patients not taking a lipid-lowering medication.**

| **Traits** | **SNP** | **Gene** | **Chr.** | **Major/minor allele** | **Model 1** | | **Model 2** | |
| --- | --- | --- | --- | --- | --- | --- | --- | --- |
|  |  |  |  |  | ***β*(SE)** | ***P*** | ***β*(SE)** | ***P*** |
| **TG** | rs3890182 | *ABCA1* | 9 | G/A | -0.040 (0.014) | **3.89×10^-3^** | -0.037 (0.014) | **6.83×10^-3^** |
|  | rs10889353 | *DOCK7* | 1 | A/C | -0.011 (0.009) | 2.19×10^-1^ | -0.012 (0.009) | 2.00×10^-1^ |
|  | rs157580 | *TOMM40* | 19 | G/A | 0.016 (0.007) | **2.54×10^-2^** | 0.015 (0.007) | **2.89×10^-2^** |
|  | rs780094 | *GCKR* | 2 | A/G | -0.017 (0.007) | **1.58×10^-2^** | -0.016 (0.007) | **1.60×10^-2^** |
|  | rs2650000 | *HNF1A* | 12 | G/T | 0.003 (0.007) | 6.60×10^-1^ | 0.004 (0.007) | 5.37×10^-1^ |
|  | rs1800961 | *HNF4A* | 20 | C/T | -0.006 (0.029) | 8.29×10^-1^ | -0.006 (0.028) | 8.31×10^-1^ |
|  | rs2240466 | *BAZ1B* | 7 | C/T | -0.039 (0.010) | **1.24×10^-4^** | -0.042 (0.010) | **2.76×10^-5^** |
| **TC** | rs3890182 | *ABCA1* | 9 | G/A | -0.006 (0.005) | 2.03×10^-1^ | -0.006 (0.005) | 2.48×10^-1^ |
|  | rs10889353 | *DOCK7* | 1 | A/C | 0.001 (0.003) | 8.75×10^-1^ | 0.001 (0.003) | 7.76×10^-1^ |
|  | rs157580 | *TOMM40* | 19 | G/A | -0.003 (0.003) | 2.32×10^-1^ | -0.003 (0.003) | 1.90×10^-1^ |
|  | rs780094 | *GCKR* | 2 | A/G | -0.005 (0.003) | 5.75×10^-2^ | -0.004 (0.003) | 8.07×10^-2^ |
|  | rs2650000 | *HNF1A* | 12 | G/T | 0.000 (0.003) | 9.61×10^-1^ | -0.000 (0.003) | 9.91×10^-1^ |
|  | rs1800961 | *HNF4A* | 20 | C/T | -0.013 (0.010) | 1.91×10^-1^ | -0.014 (0.010) | 1.69×10^-1^ |
|  | rs2240466 | *BAZ1B* | 7 | C/T | -0.006 (0.004) | 9.24×10^-2^ | -0.007 (0.004) | 6.91×10^-2^ |
| **HDL-C** | rs3890182 | *ABCA1* | 9 | G/A | -0.003 (0.006) | 6.53×10^-1^ | -0.004 (0.006) | 5.76×10^-1^ |
|  | rs10889353 | *DOCK7* | 1 | A/C | -0.004 (0.004) | 3.84×10^-1^ | -0.004 (0.004) | 3.76×10^-1^ |
|  | rs157580 | *TOMM40* | 19 | G/A | 0.000 (0.003) | 9.17×10^-1^ | 0.001 (0.003) | 8.69×10^-1^ |
|  | rs780094 | *GCKR* | 2 | A/G | -0.002 (0.003) | 5.42×10^-1^ | -0.002 (0.003) | 5.31×10^-1^ |
|  | rs2650000 | *HNF1A* | 12 | G/T | -0.005 (0.003) | 1.14×10^-1^ | -0.005 (0.003) | 9.64×10^-2^ |
|  | rs1800961 | *HNF4A* | 20 | C/T | -0.016 (0.013) | 2.39×10^-1^ | -0.015 (0.013) | 2.41×10^-1^ |
|  | rs2240466 | *BAZ1B* | 7 | C/T | -0.001 (0.005) | 7.84×10^-1^ | -0.001 (0.005) | 9.05×10^-1^ |
| **LDL-C** | rs3890182 | *ABCA1* | 9 | G/A | -0.009 (0.009) | 3.23×10^-1^ | -0.008 (0.009) | 3.94×10^-1^ |
|  | rs10889353 | *DOCK7* | 1 | A/C | 0.007 (0.006) | 2.30×10^-1^ | 0.007 (0.006) | 2.25×10^-1^ |
|  | rs157580 | *TOMM40* | 19 | G/A | -0.005 (0.005) | 2.50×10^-1^ | -0.006 (0.005) | 2.12×10^-1^ |
|  | rs780094 | *GCKR* | 2 | A/G | -0.001 (0.005) | 8.24×10^-1^ | -0.001 (0.005) | 8.19×10^-1^ |
|  | rs2650000 | *HNF1A* | 12 | G/T | -0.002 (0.005) | 6.38×10^-1^ | -0.002 (0.005) | 7.14×10^-1^ |
|  | rs1800961 | *HNF4A* | 20 | C/T | -0.025 (0.019) | 1.96×10^-1^ | -0.025 (0.019) | 1.95×10^-1^ |
|  | rs2240466 | *BAZ1B* | 7 | C/T | 0.001 (0.007) | 9.26×10^-1^ | 0.000 (0.007) | 9.76×10^-1^ |

Abbreviations: BMI, body mass index; Chr, chromosome; HDL-C, high-density lipoprotein cholesterol; LDL-C, low-density lipoprotein cholesterol; SE, standard error; SNP, single nucleotide polymorphism; TC, total cholesterol; TG, triglycerides.

All non-Gaussian distributed quantitative traits were natural logarithmically transformed to normalize distributions. *β* value and SE were determined for the minor allele of each SNP using linear regression under an additive assumption using the following models: model 1, adjusted for age and sex; model 2, adjusted for age, sex, and BMI.

*P* values <0.05 are shown in bold.
