# Supplementary material for: Genetic Variants Associated with Lipid Profiles in Chinese Patients with Type 2 Diabetes
Source: PLoS One. 2015 Aug 7;10(8):e0135145. doi: 10.1371/journal.pone.0135145 (PMC4529182; doi:10.1371/journal.pone.0135145)
Supplement: S3 Table — Abbreviations: BMI, body mass index; Chr, chromosome; HOMA-B, the homeostasis model assessment for β-cell function; HOMA-IR, the homeostasis model assessment for insulin resistance; OGTT, oral glucose tolerance test; SE, standard error; SNP, single nucleotide polymorphism. All non-Gaussian distributed quantitative traits were natural logarithmically transformed to normalize distributions. β values were calculated for the minor allele using linear regression under an additive assumption using the following models: model 1, adjusted for age and sex; model 2, adjusted for age, sex, and BMI. Associations with a P value <0.05 are shown in the table and denoted in bold. (DOCX) [file pone.0135145.s003.docx]

**S3 Table. Associations between SNPs and glycemic-related traits among newly diagnosed Chinese T2D patients not taking a lipid-lowering medication.**

| **Traits** | **SNP** | **Gene** | **Chr.** | **Minor allele** | **Model 1** | | **Model 2** | |
| --- | --- | --- | --- | --- | --- | --- | --- | --- |
|  |  |  |  |  | ***β*(SE)** | ***P*** | ***β*(SE)** | ***P*** |
| **Fasting plasma glucose** | rs780094 | *GCKR* | 2 | G | 0.008 (0.004) | **3.52×10^-2^** | 0.008 (0.004) | **3.49×10^-2^** |
| **Fasting serum insulin** | rs1800961 | *HNF4A* | 20 | T | -0.057 (0.031) | 6.76×10^-2^ | -0.064 (0.030) | **3.49×10^-2^** |
| **120-min OGTT insulin** | rs2240466 | *BAZ1B* | 7 | T | 0.040 (0.017) | **1.81×10^-2^** | 0.033 (0.016) | **4.14×10^-2^** |
| **HOMA-B** | rs1800961 | *HNF4A* | 20 | T | -0.081 (0.043) | 5.90×10^-2^ | -0.086 (0.042) | **4.06×10^-2^** |
| **HOMA-IR** | rs10889353 | *DOCK7* | 1 | C | 0.020 (0.012) | 8.21×10^-2^ | 0.022 (0.011) | **4.92×10^-2^** |

Abbreviations: BMI, body mass index; Chr, chromosome; HOMA-B, the homeostasis model assessment for β-cell function; HOMA-IR, the homeostasis model assessment for insulin resistance; OGTT, oral glucose tolerance test; SE, standard error; SNP, single nucleotide polymorphism.

All non-Gaussian distributed quantitative traits were natural logarithmically transformed to normalize distributions. *β* values were calculated for the minor allele using linear regression under an additive assumption using the following models: model 1, adjusted for age and sex; model 2, adjusted for age, sex and BMI.

Associations with a *P* value <0.05 are shown in the table and denoted in bold.
