# Supplementary material for: Genetic Variants Associated with Lipid Profiles in Chinese Patients with Type 2 Diabetes
Source: PLoS One. 2015 Aug 7;10(8):e0135145. doi: 10.1371/journal.pone.0135145 (PMC4529182; doi:10.1371/journal.pone.0135145)
Supplement: S4 Table — Abbreviations: BMI, body mass index; Chr, chromosome; DMS, Chinese National Diabetes and Metabolic Disorders Study; CI, confidence interval; OR, odds ratio; SNP, single nucleotide polymorphism. The DMS population consisted of 5,338 T2D patients and 4,663 controls as previously described [36,37]. ORs and 95% CIs were calculated for the minor allele of each SNP using logistic regression under an additive assumption using the following models: model 1, adjusted for age and sex; model 2, adjusted for age, sex, and BMI. (DOCX) [file pone.0135145.s004.docx]

**S4 Table. Associations between lipid-related SNPs and T2D in DMS patients.**

| **SNP** | **Gene** | **Chr** | **Major/minor** | **Model 1** | | **Model 2** | |
| --- | --- | --- | --- | --- | --- | --- | --- |
|  |  |  | **allele** | **OR (95%CI)** | ***P*** | **OR (95%CI)** | ***P*** |
| rs3890182 | *ABCA1* | 9 | G/A | 1.11(0.98,1.25) | 1.05×10^-1^ | 1.13(0.99,1.29) | 8.19×10^-2^ |
| rs10889353 | *DOCK7* | 1 | A/C | 0.99(0.91,1.07) | 7.29×10^-1^ | 1.00(0.92,1.09) | 9.38×10^-1^ |
| rs157580 | *TOMM40* | 19 | G/A | 1.04(0.98,1.11) | 1.63×10^-1^ | 1.05(0.99,1.12) | 1.21×10^-1^ |
| rs780094 | *GCKR* | 2 | A/G | 1.05(1.00,1.12) | 7.30×10^-2^ | 1.06(0.99,1.13) | 7.83×10^-2^ |
| rs2650000 | *HNF1A* | 12 | G/T | 0.98(0.92,1.04) | 4.42×10^-1^ | 0.96(0.90,1.02) | 1.66×10^-1^ |
| rs1800961 | *HNF4A* | 20 | C/T | 1.06(0.85,1.32) | 5.98×10^-1^ | 1.06(0.83,1.35) | 6.22×10^-1^ |
| rs2240466 | *BAZ1B* | 7 | C/T | 1.00(0.92,1.09) | 9.72×10^-1^ | 0.96(0.87,1.05) | 3.56×10^-1^ |

Abbreviations: BMI, body mass index; Chr, chromosome; DMS, Chinese National Diabetes and Metabolic Disorders Study; CI, confidence interval; OR, odds ratio; SNP, single nucleotide polymorphism.

The DMS population consisted of 5,338 T2D patients and 4,663 controls as previously described [36,37]. ORs and 95% CIs were calculated for the minor allele of each SNP using logistic regression under an additive assumption using the following models: model 1, adjusted for age and sex; model 2, adjusted for age, sex, and BMI.
